# Supplementary material for: Reproductive characteristics and gametogenic cycle of the scleractinian coral Dendrophyllia ramea
Source: PeerJ. 2023 Sep 29;11:e16079. doi: 10.7717/peerj.16079 (PMC10544315; doi:10.7717/peerj.16079)
Supplement: Supplemental Information 1 — The year season of each sample, specific sampling date, location, depth range and number of collected colonies. [file peerj-11-16079-s001.docx]

**SUPPLEMENTARY MATERIAL 1: SAMPLES COLLECTED**

**Suppl Mat 1: collected samples of *Dendrophyllia ramea*.**

Table includes: the year season of each sample, specific sampling date, location, depth range and number of collected colonies.

| **Year season** | **Month** | **Date** | **Location** | **Depth (m)** | **Sample/colony** |
| --- | --- | --- | --- | --- | --- |
| Winter | February | 03.02.2018 | Punta La Mona | 36.5 | 4 |
| Winter | February | 03.02.2018 | Punta La Mona | 36.5 | 12 |
| Winter | February | 03.02.2018 | Punta La Mona | 36.5 | 13 |
| Winter | February | 03.02.2018 | Punta La Mona | 36.5 | 14 |
| Winter | February | 03.02.2018 | Punta La Mona | 36.5 | 17 |
| Winter | February | 03.02.2018 | Punta La Mona | 36.5 | 22 |
| Winter | February | 03.02.2018 | Punta La Mona | 36.5 | 28 |
| Winter | February | 03.02.2018 | Punta La Mona | 36.5 | 42 |
| Winter | February | 03.02.2018 | Punta La Mona | 36.5 | 47 |
| Winter | February | 03.02.2018 | Punta La Mona | 36.5 | 58 |
| Spring | May | 27.05.2017 | Punta La Mona | 36 | 25 |
| Spring | May | 27.05.2017 | Punta La Mona | 36 | 26 |
| Spring | May | 27.05.2017 | Punta La Mona | 36 | 33 |
| Spring | May | 27.05.2017 | Punta La Mona | 36 | 35 |
| Spring | May | 27.05.2017 | Punta La Mona | 36 | 41 |
| Spring | May | 27.05.2017 | Punta La Mona | 37 | 49 |
| Spring | May | 27.05.2017 | Punta La Mona | 36 | 50 |
| Summer | June | 24.06.2017 | Punta La Mona | 34-36 | 2 |
| Summer | June | 24.06.2017 | Punta La Mona | 34-36 | 9 |
| Summer | June | 24.06.2017 | Punta La Mona | 34-36 | 23 |
| Summer | June | 24.06.2017 | Punta La Mona | 34-36 | 24 |
| Summer | June | 24.06.2017 | Punta La Mona | 34-36 | 27 |
| Summer | June | 24.06.2017 | Punta La Mona | 34-36 | 39.9 |
| Summer | June | 24.06.2017 | Punta La Mona | 34.9 | 54 |
| Summer | June | 24.06.2017 | Punta La Mona | 34.9 | 55 |
| Summer | June | 24.06.2017 | Punta La Mona | 34.9 | 56 |
| Summer | June | 24.06.2017 | Punta La Mona | 34.9 | 57 |
| Summer | July | 29.07.2018 | Punta La Mona | 33.3 | 5 |
| Summer | July | 29.07.2018 | Punta La Mona | 33.3 | 7 |
| Summer | July | 29.07.2018 | Punta La Mona | 33.3 | 15 |
| Summer | July | 29.07.2018 | Punta La Mona | 33.3 | 21 |
| Summer | July | 29.07.2018 | Punta La Mona | 33.3 | 32 |
| Summer | July | 29.07.2018 | Punta La Mona | 33.3 | 34 |
| Summer | July | 29.07.2018 | Punta La Mona | 33.3 | 39 |
| Summer | July | 29.07.2018 | Punta La Mona | 33.3 | 40 |
| Summer | July | 29.07.2018 | Punta La Mona | 33.3 | 48 |
| Summer | July | 29.07.2018 | Punta La Mona | 33.3 | 52 |
| Autum | October | 02.10.2017 | Punta La Mona | 34-36 | 19 |
| Autum | October | 02.10.2017 | Punta La Mona | 34-36 | 20 |
| Autum | October | 02.10.2017 | Punta La Mona | 34-36 | 30 |
| Autum | October | 02.10.2017 | Punta La Mona | 34-36 | 31 |
| Autum | October | 02.10.2017 | Punta La Mona | 34-36 | 36 |
| Autum | October | 02.10.2017 | Punta La Mona | 34-36 | 37 |
| Autum | October | 02.10.2017 | Punta La Mona | 34-36 | 38 |
| Autum | October | 02.10.2017 | Punta La Mona | 34-36 | 45 |
| Autum | October | 02.10.2017 | Punta La Mona | 34-36 | 46 |
| Autum | October | 02.10.2017 | Punta La Mona | 34-36 | 51 |
| Autum | October | 02.10.2017 | Punta La Mona | 34-36 | 53 |
| Autum | November | 10.11.2017 | Punta La Mona | 34-36 | 1 |
| Autum | November | 10.11.2017 | Punta La Mona | 34-36 | 3 |
| Autum | November | 10.11.2017 | Punta La Mona | 34-36 | 6 |
| Autum | November | 10.11.2017 | Punta La Mona | 34-36 | 8 |
| Autum | November | 10.11.2017 | Punta La Mona | 34-36 | 10 |
| Autum | November | 10.11.2017 | Punta La Mona | 34-36 | 11 |
| Autum | November | 10.11.2017 | Punta La Mona | 34-36 | 16 |
| Autum | November | 10.11.2017 | Punta La Mona | 34-36 | 18 |
| Autum | November | 10.11.2017 | Punta La Mona | 34-36 | 29 |
| Autum | November | 10.11.2017 | Punta La Mona | 34-36 | 43 |
